# Cryptococcus neoformans Periprosthetic Joint Infection in the Setting of Revision Shoulder Arthroplasty: A Case Report

**DOI:** 10.7759/cureus.71253

**Published:** 2024-10-11

**Authors:** Speros Gabriel, Michael A Boin

**Affiliations:** 1 Orthopedic Surgery, HCA Research Medical Center, Kansas City, USA; 2 Orthopedic Surgery, North Kansas City Hospital, Kansas City, USA

**Keywords:** anti-microbial resistance, cryptococcus neoformans (c. neoformans), orthopedic infectious disease, peri-prosthetic joint infection, revision shoulder arthroplasty

## Abstract

An 83-year-old male, status one year post-primary right reverse shoulder arthroplasty, presented with clinical and radiographic signs of shoulder instability. The patient subsequently underwent revision reverse shoulder arthroplasty with postoperative cultures growing *Cryptococcus neoformans* in all five tissue samples. He was placed on temporary fluconazole since he demanded to leave the hospital during a holiday weekend with plans to get readmitted later. IV amphotericin B was used as induction treatment for two weeks followed by PO voriconazole, which was taken by the patient until eight months postoperatively. He was followed up in the orthopedic clinic status post revision procedure at one and two months and was found to be progressing well.

The patient presented to the emergency department eight months after the revision procedure for an unrelated concern that was inconsequential. There were no signs of infection as vital signs and laboratory markers were normal. At 18 months postoperatively, he presented to an outside facility with a left distal femur periprosthetic fracture around a total knee implant, for which open reduction and internal fixation (ORIF) was performed. During this hospital stay, an orthopedic team, known to the authors, evaluated the patient. The right shoulder was stable and without pain, with vitals signs and laboratory markers showing no signs of infection. The patient reported that he continued to be employed as a maintenance worker at a motel. He was discharged soon and is living with current revision shoulder arthroplasty implants.

## Introduction

Periprosthetic joint infection (PJI) is one of the most prevalent reasons for revision shoulder surgery, with Boileau et al. citing it as the second most common reason for revision surgery [1-3]. The most commonly identified pathogens in periprosthetic shoulder infections include *Cutibacterium acnes*, *Staphylococcus epidermidis,* and *Staphylococcus aureus* [1-3]. PJIs caused by fungal species are much rarer. Fungal sources of PJI account for 1-2% of all periprosthetic infections [4]. The majority of fungal PJIs are due to *Candida albicans* [4]. Shoulder arthroplasty PJI due to cryptococcus is exceedingly rare. A literature search revealed only three case reports that described a PJI due to cryptococcal infection and only one of them involved shoulder arthroplasty [4-6]. 

In 2022, the World Health Organization (WHO) released a fungal priority pathogens list of fungal species that require more attention due to their high resistance to treatment, increased mortality rates, and lack of consistent treatment guidelines [7-9]. *Cryptococcus neoformans* is included in the “critical” category, emphasizing the critical need for further research on this pathogen [7-8]. Cryptococcus is an opportunistic encapsulated yeast with a global presence [7,10]. The fungus can be found most commonly in bird feces, soil, and decaying wood [8,11]. Being an opportunistic pathogen, Cryptococcus usually infects immunocompromised individuals such as those with HIV, T-cell disorders, and organ transplant recipients, among others [8]. 

The usual pathogenesis involves inhalation of basidiospores or desiccated yeast cells through the respiratory system, leading to pneumonitis and pneumonia in immunocompromised but also asymptomatic latent infection in immunocompetent patients [8]. The fungal cells can then disseminate to other locations through hematogenous spread [4,7,12]. The most common location of dissemination is the central nervous system (CNS), resulting in meningitis and meningoencephalitis [11]. Cryptococcus can also be present in the skin as cutaneous cryptococcus in the setting of a skin wound [8]. The usual presentation of infections related to cryptococcus does not entail primary osteoarticular involvement, with infections involving the skeletal system comprising <10% [4,12]. While treatment guidelines are available for the management of more common forms of cryptococcal infections in the literature, there is a lack of well-defined treatment algorithms for cryptococcal PJI [9]. We present a rare case of *Cryptococcus neoformans* PJI in the setting of revision shoulder arthroplasty.

## Case presentation

**Figure 1:**
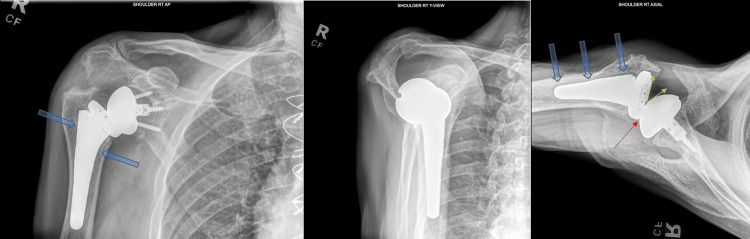
Preoperative radiographs of the right shoulder The images show anterior humeral subluxation (green arrowed v-sign demonstrating increased space at the prosthesis articulation anteriorly with loss of concentric alignment of the humeral component on the glenosphere); posterior metal-on-metal component contact of the humeral and glenoid components (red thin arrow); and slight lucency around the humeral component (blue thick arrows)

The patient was an 83-year-old male with a past medical history of coronary artery disease status post-coronary artery bypass grafting, abdominal aortic aneurysm status post-stenting, thoracic aortic aneurysm, transient ischemic attack, atrial flutter, peripheral vascular disease with lower extremity thrombectomy and stent placement, diabetes, and bilateral total knee arthroplasty. There was no previous history of immunocompromise. He had undergone primary right reverse shoulder arthroplasty for rotator cuff arthropathy, by another physician, at the age of 80. He presented to our clinic 12 months after this procedure for a painful right shoulder, reporting pain and dysfunction since the time of the index reverse shoulder arthroplasty. Radiographic evaluation of the shoulder revealed anterior humeral subluxation, posterior metal-on-metal component contact of the humeral and glenoid components, and slight lucency of the humeral component (Figure 1).

The components were determined to be the LINK system. Revision surgery was recommended for instability and revision reverse shoulder arthroplasty was performed two months after this visit. Preoperative laboratory results were negative for any signs of infection. This procedure was uneventful and the patient was discharged on postoperative day (POD) two. However, he was readmitted on POD three due to postoperative tissue cultures growing *Cryptococcus neoformans*. Infectious disease (ID) was consulted, and recommendations were provided in coordination with medicine and orthopedic services. Fluconazole 400 mg PO was given from POD three to POD seven, as the patient demanded to leave the hospital over the holiday weekend, even though the ID service recommended IV amphotericin B.

The patient was readmitted to the hospital on POD eight and IV amphotericin B 350 mg daily was started. He completed a 14-day course of IV amphotericin B and was then transitioned to PO voriconazole 200 mg BID with recommendations for lifetime dosing. Minimal inhibitory concentration results returned as an intermediate grade for fluconazole use against the cultured* Cryptococcus neoformans*, which prompted the selection of voriconazole for treatment. All five intraoperative cultures were positive for *Cryptococcus neoformans. *A two-stage revision procedure was discussed with the patient after the return of all cultures. Given the absence of postoperative signs of infection, treatment with suppressive antibiotics was initiated. 

**Figure 2:**
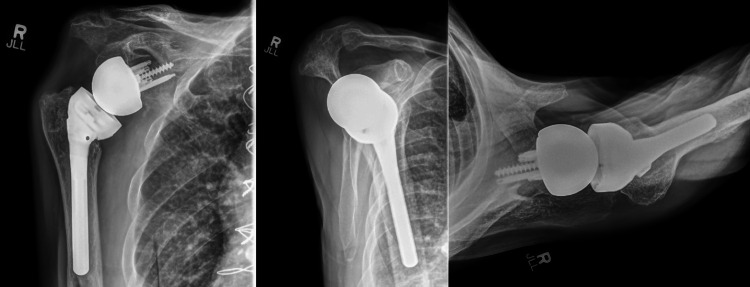
Radiographs of the right shoulder at the four-week postoperative visit demonstrating well-aligned revision reverse shoulder arthroplasty implants

**Figure 3:**
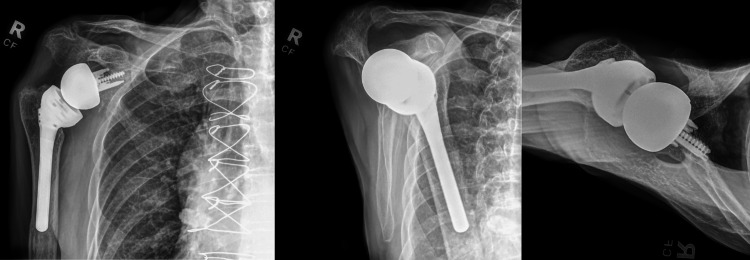
Radiographs of the right shoulder at the 9.5-week postoperative visit demonstrating well-aligned revision reverse shoulder arthroplasty implants

At the one-month postoperative orthopedic follow-up, the patient was doing well with shoulder range of motion as expected and intact neurovascular status. He was seen again in the orthopedic clinic at 9.5 weeks postoperatively. At this visit, the shoulder's active range of motion was 140 degrees of flexion, 40 degrees of external rotation, and internal rotation to the hip pocket. Labs drawn 10 days before this encounter had demonstrated a normal C-reactive protein (CRP) of <5.0 mg/dL (reference range: 0-10) and an erythrocyte sedimentation rate (ESR) of 28 mm/hr (reference range: 0-17). Radiographs at both clinic visits demonstrated well-fixed implants in appropriate alignment (Figures 2-3).

The patient again presented to the emergency department, at the same hospital, at around eight months status post-revision shoulder procedure for an unrelated concern that was inconsequential. He did not report shoulder discomfort, per documentation. Vital signs during this ER visit included a Tmax of 97.9 °F and a heart rate (HR) of 65 beats per minute (BPM). Laboratory testing demonstrated a hemoglobin of 11.4 g/dL, white blood cell (WBC) count of 10.6 K/µL, platelet count (PLT) of 180 K/µL, and lactate of 1.1 mmol/ L. He was recommended to continue with aspirin and Eliquis home dosing. The patient was followed up by the treating infectious disease physician in the outpatient setting and was confirmed to be taking the prescribed voriconazole until eight months postoperatively. He opted to discontinue voriconazole due to the high price of the medication, although the infectious disease team did encourage the continuation of the medication for life. At 15 months postoperatively, the patient had labs drawn with a CD4 count of 1071.0 cells/µL on a scale of (reference range: 490-1600).

At 18 months postoperatively, the patient presented to an outside facility with a left distal femur periprosthetic fracture. He subsequently underwent open reduction and internal fixation (ORIF) for this injury. During the hospital stay, the outside orthopedic team evaluated the patient and documented that the right shoulder was not painful, the range of motion was intact, the skin was intact, and the hand was functioning properly. This orthopedic team was known to the authors and the patient evaluation was discussed with one of the providers. The patient had stated that he continued to be employed as a maintenance worker for a motel and was functionally active. Vital signs demonstrated a Tmax of 97.9 °F, HR of 60 BPM, blood pressure of 105/76 mmHg, respiratory rate of 19-28 breaths per minute, and O_2_ sat. of 98%. Laboratory results showed a hemoglobin of 11.7 g/dL, and WBC counts of 14.95k cells/µL (repeat WBC four days later with 9.9k cells/µL). Vascular surgery was consulted due to complications of peripheral vascular disease. The patient was able to use a rolling walker with toe-touch weight-bearing to the left lower extremity. He was discharged to home on postoperative day two. At the time of article submission, the patient is living with the current revision arthroplasty implants.

## Discussion

Cryptococcal PJI is an exceedingly rare complication with only three reported cases in the literature to our knowledge, with only one of them relating to a shoulder prosthesis [4-6].The management of reported cases has included consideration of revision procedure, prolonged antifungal course, and lifelong antifungal therapy [4-6,12]. Johannsson et al. published the first case report of PJI due to a cryptococcus species [5]. The authors reported the case of an 84-year-old patient with a left revision hip PJI with cultures of joint fluid growing *Cryptococcus neoformans* and pre-fungal treatment radiographs demonstrating peri-implant osteolysis around revision components. The patient had a previous left total hip arthroplasty (THA) PJI due to methicillin-resistant *Staphylococcus aureus *(MRSA)*, *resulting in an exchange of implants nine years prior. The *Cryptococcus neoformans* PJI of revision implants was treated with amphotericin B deoxycholate for 12 days (total dose of 800 mg), followed by a transition to long-term fluconazole (400 mg/day). The revision THA implants were removed at around 10 months postoperatively due to worsening pain, continued peri-implant osteolysis, and secondary acetabular protrusio of the metal cup.

Baptista et al. have reported a case of a patient with a shoulder cement spacer demonstrating polymerase chain reaction and DNA sequencing positive for *Cryptococcus magnus* joint infection [4]. This involved a revision cement spacer after the patient had a primary PJI around an anatomic total shoulder arthroplasty (TSA) that grew Methicillin-resistant *Staphylococcus epidermidis* (MRSE). The TSA PJI was originally treated with debridement, antimicrobial therapy, and implant retention (DAIR) with subsequent conversion to cement antibiotic spacer, which eventually grew MRSE. This spacer was then converted to the final spacer that was colonized with *Cryptococcus magnus *and remains as the patient’s current implant. The patient’s* Cryptococcus magnus* PJI was managed successfully with two weeks of IV amphotericin B followed by six weeks of fluconazole and remains with the revision cement spacer. Shah et al. reported a case of a 77-year-old woman with bilateral THA who underwent a unilateral two-stage revision hip procedure for coagulase-negative staphylococcal PJI [6]. The patient developed *Cryptococcus neoformans* PJI around the revision hip construct with cultures from hematoma and capsule growing *Cryptococcus neoformans*. The patient was treated with an initial course of daily IV amphotericin (4mg/kg) for 12 weeks with a transition to long-term fluconazole (200 mg daily).

Antifungal treatment for invasive fungal diseases falls into four categories: azoles, polyenes, pyrimidines, and echinocadens [8]. Cryptococcus species are intrinsically resistant to echinocadens and have demonstrated the ability to develop resistance to azoles [8-9]. This leaves three main classes of antifungals, with several articles published in the last 10 years reporting a growing trend of cryptococcus resistance to azoles [9,13-15]. Cryptococcus has demonstrated early resistance to Flucytosine, a pyrimidine, when used in isolation [9]. Amphotericin, a polyene, has been the drug of choice for induction therapy of Cryptococcal CNS infection, disseminated disease, and severe pulmonary infection [8-9]. Mild pulmonary infection may be treated with isolated fluconazole, according to Chang et al [9]. Clinical practice guidelines regarding Cryptococcal infection relating to isolated skin infection or disseminated disease to a singular anatomic location favor an extended course of fluconazole, with Perfect et al. recommending 6-12 months of treatment and a more recent review article by Chang et al. (2024) recommending three to four months of treatment [9,12]. The correlation of these recommendations to periprosthetic infection remains unknown. There are currently no clinical trials on the management of non-CNS, non-pulmonary cryptococcal infection [9].

Our report demonstrates the successful management of a revision shoulder arthroplasty following unexpected positive cultures of *Cryptococcus neoformans. *This was managed with antifungal therapy and the retention of implants. The authors' preferred management of unexpected positive cultures involves a single-stage procedure with antimicrobial therapy. A two-stage revision was discussed with the patient as a possible option following positive cultures. However, due to a lack of signs of any postoperative infection, suppressive antifungal medication was the chosen treatment. The patient was evaluated clinically and radiographically by the operating surgeon at four and 9.5 weeks postoperatively, with the patient progressing as expected. ESR and CRP were obtained at around two months postoperatively, with the CRP in the normal range and the ESR appropriately mildly elevated after a surgical procedure.

We were able to evaluate vital signs and laboratory markers at eight and 18 months postoperatively through an ER presentation for an unrelated concern and an outside hospital presentation for left distal femur periprosthetic ORIF, respectively. The patient had a normal Tmax and HR, as well as reassuring laboratory markers revealing low concern for systemic inflammatory response syndrome at both visits. The patient did not report shoulder pain at either presentation. Voriconazole was taken by the patient for eight months after the revision procedure despite a lifetime treatment recommendation by the infectious disease team. At the time of submission (18 months postoperatively), the patient is known to be living with the retained revision shoulder implants.

## Conclusions

We believe the findings of this report are of immense value to the medical community, given the threat of increasing cryptococcus resistance to current treatment methods, known cases of primary cryptococcal infection in immunocompetent individuals, and a general lack of treatment algorithms for cryptococcal PJI.This case highlights a rare *Cryptococcus neoformans* periprosthetic shoulder joint infection of revision arthroplasty components. The management included the retention of implants with an initial course of IV amphotericin B and a transition to PO voriconazole, with the medication being taken for eight months after the revision procedure. The patient was known to be stable through 18 months after insertion of revision shoulder arthroplasty implants.

Our literature review revealed that revision arthroplasty is the setting in which cryptococcus has been cultured in the presence of prosthetic implants. This report presents the first case where primary arthroplasty implants were seeded with *Cryptococcus neoformans*, and the revision components were exposed to the pathogen. In light of numerous studies predicting the exponential increase in primary and revision arthroplasty procedures being performed and the possibility of fungal resistance to current treatment methods, further research is recommended to devise optimal management options for cryptococcal PJI.
